# Epidemiological analysis of confirmed mpox cases, Burundi, 3 July to 9 September 2024

**DOI:** 10.2807/1560-7917.ES.2024.29.42.2400647

**Published:** 2024-10-17

**Authors:** Alexis Nizigiyimana, Francois Ndikumwenayo, Sarah Houben, Martin Manirakiza, Monique van Lettow, Laurens Liesenborghs, Placide Mbala-Kingebeni, Anne W Rimoin, Isaac I Bogoch, Jason Kindrachuk

**Affiliations:** 1 Department of Management, Evaluation and Health Policy, School of Public Health, Université de Montréal, Montreal, Canada; 2 These authors contributed equally.; 3 University of Burundi, Bujumbura, Bujumbura, Burundi; 4 Department of Clinical Sciences, Institute of Tropical Medicine, Antwerp, Belgium; 5 Burundi NCD Alliance, Bujumbura, Burundi; 6 Madiro, Toronto, Canada; 7 Dalla Lana School of Public Health, University of Toronto, Toronto, Canada; 8 Institut National de Recherche Biomédicale (INRB), Kinshasa, Democratic Republic of the Congo; 9 Service de Microbiologie, Département de Biologie Médicale, Cliniques Universitaires de Kinshasa, Université de Kinshasa, Kinshasa, Democratic Republic of the Congo; 10 Fielding School of Public Health, University of California Los Angeles, Los Angeles, United States; 11 Department of Medicine, University of Toronto, Toronto, Canada; 12 Department of Medical Microbiology and Infectious Diseases, University of Manitoba, Winnipeg, Canada; 13 Department of Internal Medicine, University of Manitoba, Winnipeg, Canada

**Keywords:** mpox, monkeypox virus, Clade Ib, Burundi, epidemiology, hospitalisation

## Abstract

We analysed mpox cases in Burundi from July to September 2024, following the introduction of Clade Ib virus. Of 607 samples from the whole population of suspected cases, 154 were PCR-positive, of whom 85 were children under 15 years, with a higher proportion of female children testing positive. Geographical analysis demonstrates case concentration in Bujumbura Mairie (91/154). Age- and sex-specific interventions, as well as community engagement, are important for outbreak containment, as are targeted public health strategies in Burundi.

From January 2023 to present, broad geographic expansion of Clade I MPXV in the Democratic Republic of the Congo (DRC) has resulted in the largest mpox outbreak thus far recorded among all endemic countries. This national outbreak has included the expansion of MPXV to all provinces in DRC as well as the emergence of a new virus subclade, Clade Ib, associated with sustained human-to-human transmission [1]. While first reported in South Kivu province, DRC, Clade Ib has undergone rapid regional expansion that has included introduction to North Kivu, DRC [2], as well as dissemination to neighbouring countries in East Africa. Case totals were highest in Burundi with 987 confirmed cases as of 13 October 2024.

Here, we provide an analysis of demographic and epidemiological considerations for mpox in Burundi using mpox case data from 3 July to 9 September 2024.

## Demographic data of mpox cases in Burundi

**Figure 1:**
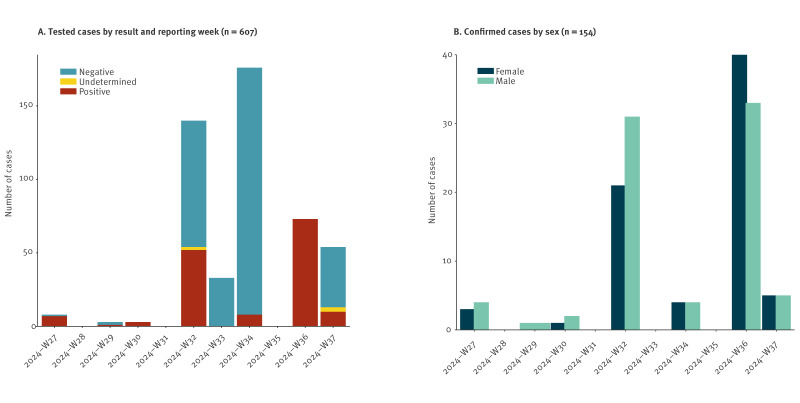
Demographic data of suspected and confirmed mpox cases, by sex, Burundi, 3 July–9 September 2024 (n = 607)

We analysed national mpox testing and demographic data provided to us by the Ministry of Health of Burundi for the period from 3 July 2024 to 9 September 2024 (week 27 to week 37). During this period, a total of 607 samples were tested by PCR for MPXV and those who tested positive were defined as confirmed cases (Figure 1 and Table 1). Of these, men comprised 305 samples (50.2%) and women comprised 302 samples (49.8%). For all samples, 154 samples (25.4%) tested positive, 448 samples (73.8%) tested negative, and five samples (0.8%) were indeterminate. Of the positive cases, males comprised 51.9% of cases (80/154) and women comprised 48.1% (74/154).

**Table 1:** Mpox PCR test results by sex, Burundi, 3 July–9 September 2024 (n = 607)

	Positive		Negative		Indeterminate	
	n	%	n	%	n	%
Male	80	13.2	223	49.8	2	0.33
Female	74	12.2	225	50.2	3	0.49
**Total**	154	25.4	448	73.8	5	0.82

**Figure 2:**
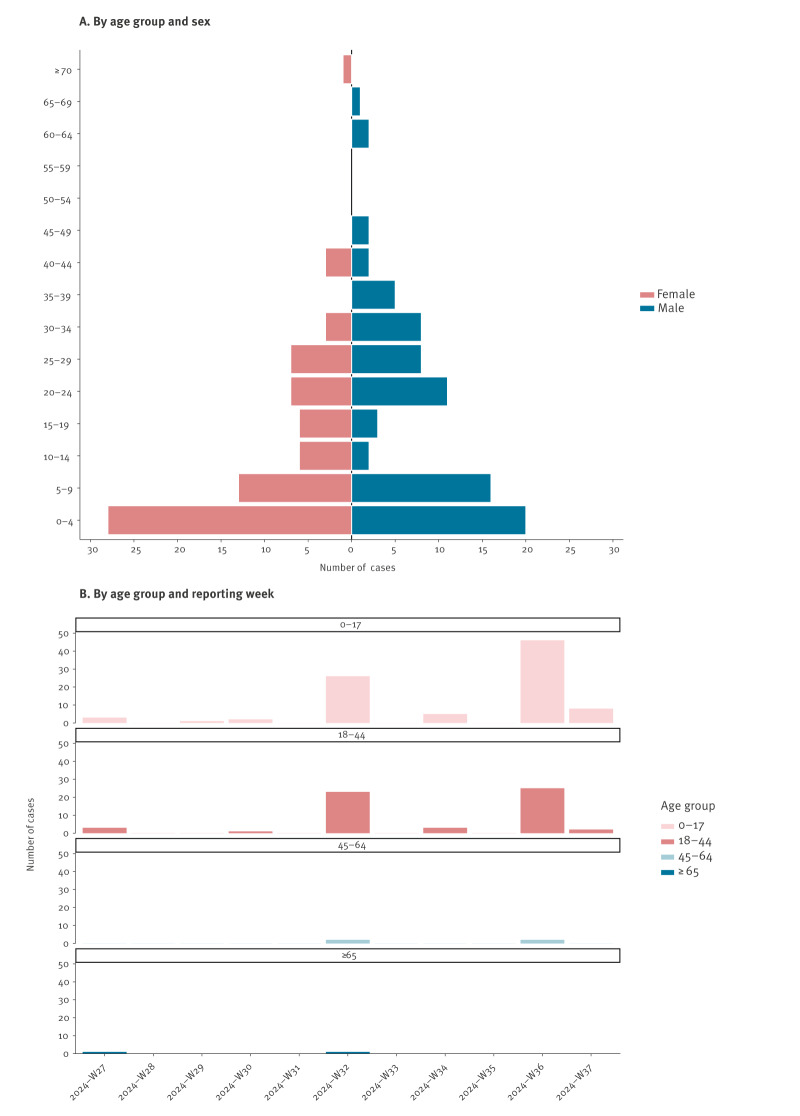
Demographic data for individuals with confirmed mpox, by age, Burundi, 3 July–9 September 2024 (n = 154)

The median age of all individuals tested was 7 years (interquartile range (IQR): 2–18 years) with an age range of < 6 months to 72 years (Figure 2). For MPXV-positive cases, the median age was 9.5 years (IQR: 3–25 years). The median age of positive cases was significantly higher for males than females at 17.5 years vs 6 years (p = 0.018). The median age of males and females tested was 8 years and 6 years, respectively (p = 0.044). Wilcoxon tests were used for all statistical analyses presented. An age pyramid for mpox cases separated by sex is presented in Figure 2A. Overall, children < 15 years comprised the majority of all positive cases (85/154; 55.2%) with cases highest among 0–4-year-olds, followed by 5–9-year-olds (Table 2).

**Table 2:** Age and sex demographics of mpox cases, Burundi, 3 July–9 September 2024 (n = 154)

		n	Age (years)		p value
			Median	IQR	
All tested	Male	302	8.0	2–15	0.044
	Female	305	6.0	2–23	
	**Total population**	**607**	**7.0**	**2–18**	**NA**
Positive	Male	80	17.5	5–29	0.018
	Female	74	6.0	2–20	
Negative	Male	223	7.0	2–17.5	0.417
	Female	225	6.0	2–14	
**Positive**	**Total population**	**154**	**9.5**	**3–25**	**0.046**
**Negative**		**448**	**6.0**	**2–15**	

IQR: interquartile range; NA: not applicable.

## Geographical distribution of mpox cases in Burundi

**Figure 3:**
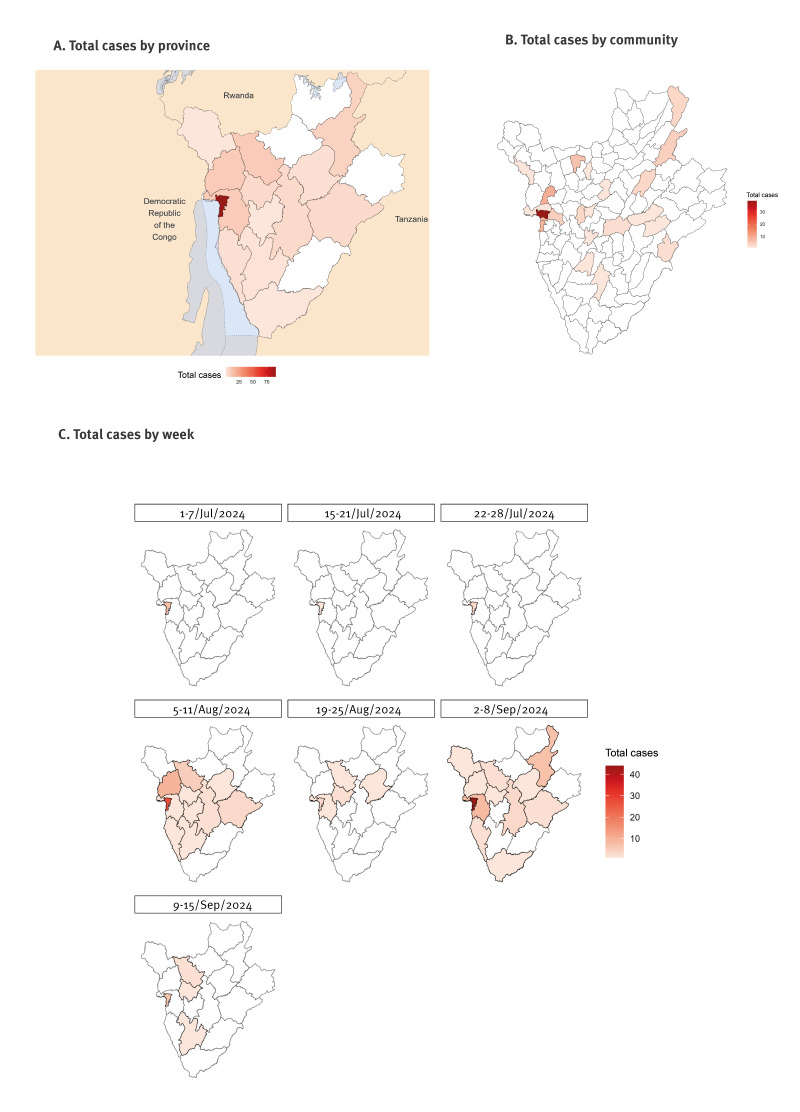
Geographical distribution of mpox cases, Burundi, 3 July–9 September 2024 (n = 154)

Confirmed mpox cases were identified from 14 provinces in Burundi (Figure 3) with the majority of PCR-positive cases identified in the province Bujumbura Mairie (91/154; 59.1%), followed by Bubanza, Bujumbura and Kayanza with 10 cases reported from each (6.5% of positive cases each). Associations between geographical region and age distribution of cases were limited due to the high proportion of overall mpox cases reported from Bujumbura Mairie and much lower proportions in any of the other provinces reporting. We did analyse age demographics in Bujumbura Mairie as compared with the complete national dataset. Of the 91 mpox cases reported in Bujumbura Mairie, children < 15 years comprised 48.4 (44/91), followed by those aged 15–30 years at 35.2% (32/91) and lastly those ≥ 30 years with 16.5% of cases (15/91). When combining positive case data from all other provinces (63 cases total), children < 15 years comprised the highest proportion with 41 cases, followed by those aged 15–30 years (13 cases) and those ≥ 30 years (nine cases).

## Mpox-associated hospitalisations and clinical symptoms

We analysed available hospitalisation data from the teaching hospital in Bujumbura where data from MPXV-positive patients were available from July up to 25 September 2024, including a total of 254 cases with 247 requiring care for more than 1 day (Table 3). The median age of those hospitalised was 19 (IQR: 8–28) with a median of 16 years (IQR: 7–25) for female and 22 years (IQR: 8–32) for male patients (p = 0.013). There was no significant difference in overall duration of hospital admission between males and females with a total median of 8 days (IQR: 6–12) for all patients and 8 days (IQR: 5–9.8) and 9 days (IQR: 6–12) for females and males, respectively. No deaths were recorded, but 45 patients remained hospitalised at the time of this report. The majority of patients had no known comorbidities. For hospitalised patients, HIV positivity was noted among seven patients, as was hypertension (seven cases), followed by asthma (five cases) and diabetes (four cases). 

**Table 3:** Hospitalisation data for mpox cases, Bujumbura hospital, Burundi, 3 July–25 September 2024 (n = 247)

	n	Age (years)		p value
		Median	IQR	
Male	132	22	8–32	0.013
Female	115	16	7–25	
**Total**	**247**	**19**	**8–28**	**NA**

IQR: interquartile range; NA: not applicable.

Hospitalisation duration unavailable for seven patients.

We also analysed clinical symptom and outcome data that were available for 249 of the 254 patients seen at Bujumbura hospital. This included seven patients with unavailable data for the duration of their hospitalisation and one patient with no data for outcome. Fever was reported for 50.4% of patients (126/250), with headache (19.6%; 49/250) and myalgia (1.2%; 3/250) reported less commonly. Lymphadenopathy was reported for 36.8% of patients (92/250). Rash was reported for nearly all patients (249/250) and with pustular or vesiculopapular rash most common (92%; 229/249). Generalised rash was reported most (78.3%; 195/249), and genital rash was reported among 19.7 of patients (49/249). Sore throat (including cough) was reported among 15.6% of patients (39/250). Nearly all hospitalised patients were reported as recovered or recovering (99.2%; 247/249); two cases of loss of vision were reported among the outcomes (0.8%), although it was not reported whether this was transient or permanent. Outcome data were not provided for one patient. 

## Discussion

Mpox is a zoonotic viral infectious disease first identified in DRC in 1970 [3-5]. Monkeypox virus (MPXV) historically circulated in the tropical forest regions of Central and West Africa, where it is considered endemic [6,7]. The virus is subclassified into two clades: clade I, formerly known as the Congo Basin (Central Africa) clade, and clade II, formerly known as the West African clade. Clade II is further subdivided into two subclades: IIa and IIb, with the latter responsible for the global epidemic in 2022 [8,9].

Historically, zoonotic transmission has been the primary driver of MPXV infections in humans with secondary infections through human-to-human contact. However, Clade IIb MPXV infections during the 2022 global epidemic were linked primarily to sustained human-to-human transmission [10] including among men who have sex with men [11]. The emergence of Clade Ib MPXV has demonstrated similar transmission characteristics including linkage to sexual networks and sustained human-to-human transmission [1]. However, Clade Ib mpox cases have not been overrepresented among males. Further, recent expansion of Clade Ib into North Kivu, DRC, has included probable transmission through non-sexual or intimate contacts, including to children. While there may be reporting biases complicating a more wholesome understanding of mpox epidemiology, containment and mitigation strategies in this ongoing public health emergency must also consider the context-dependencies of these differences.

This report provides some insights into patients infected with Clade Ib MPXV. Sex-specific differences were present when considering age, MPXV positivity and hospitalisation. While the median ages of males and females were very similar among those who tested negative for MPXV, there was a significant difference in median age between male and female MPXV-positive cases (17.5 years and 6 years, respectively), highlighting potential disparate sex-based epidemiological risk factors for infection. However, it should be noted that there was strong overlap in the IQRs for age of MPXV-positive male and female cases which thus warrants more detailed investigation. Further case investigations are needed to better understand how exposures differ among males and females in Burundi and to better inform community recommendations on infection prevention and control.

No fatalities were noted among patients hospitalised with mpox, but not all had been discharged at the time of writing this report. Generalised rash was very common among hospitalised patients, and genital rash was reported in 18.1% of patients with a median age of 23 years (IQR: 19–30). This suggests that the contributions of sexual (intimate) and non-sexual contact-mediated transmission needs further investigation in Burundi. Two reports of vision loss were included among the hospitalised patients, both in individuals ≥ 15 years. These early epidemiological data demonstrate that mpox cases during the studied period were overrepresented among children < 15 years and the highest incidence was among those ≤ 9 years. Whether these data are reflective of certain activities or behaviours associated with greater risk of MPXV infection across sex and age groups needs to be further investigated (e.g. household caregiving, contact with contaminated materials). In addition, while mpox cases in Burundi were overrepresented among children < 15 years, the median age of hospitalisation for both male and female cases was > 15 years. It is also prudent to consider recent data from North Kivu which included probable non-sexual (intimate) contact-based transmission events within and outside of households, including to children [2]. Thus, community engagement and messaging activities in Burundi should consider these early associations between MPXV positivity status, sex and age. In addition, the data provided for this study suggest that the highest incidence of MPXV-positive cases occurred in children, morbidity impacts appeared to be greatest among those ≥ 15 years. This may provide some indication for future vaccination programmes regarding target populations to reduce morbidity impacts; however, the data should be taken with caution due to many study limitations. 

There were several limitations in this study, including the inability to fully access all nationwide data for confirmed mpox cases that included matched clinical data. This resulted in differences between the confirmed mpox case data reported for our analysis of sex- and age-specific case trends and the hospitalisation data. However, the PCR-confirmed data do represent more than one region of the country and can probably provide context for nationwide trends. We do not have any additional information to infer transmission routes, linkages to travel, household-related transmission, etc from the data we were able to obtain. This includes the reasoning for the fluctuations in MPXV-positive cases reported during week 32 and week 36 (e.g. differential access to testing or case reporting). Additional case investigations are needed to inform these questions. Regarding hospitalisation data, greater access to data sources that include multiple hospitals in rural and urban regions would help provide additional context for regional differences that may exist regarding clinical symptoms and outcomes. 

## Conclusion

This report provides an early assessment of the demographic and epidemiological characteristics of Clade Ib mpox cases in Burundi and insights for ongoing outbreak containment and mitigation strategies, including community engagement and messaging activities. Given the high mpox case incidence among children < 15 years in Burundi and increasing concerns regarding further Clade Ib expansion in Africa, our data provide initial clinical insights for Clade Ib morbidity and mortality in children where there were no fatalities among children and the median age of those hospitalised was 19 years. Here, while cases have been overrepresented among those < 15 years, hospitalisation data did not show a similar trend and may suggest differences compared with Clade Ia among this age group. 

## References

[1] Vakaniaki EH Kacita C Kinganda-Lusamaki E O’Toole Á Wawina-Bokalanga T Mukadi-Bamuleka D Sustained human outbreak of a new MPXV clade I lineage in eastern Democratic Republic of the Congo. Nat Med. 2024;1(11):1. 10.1038/s41591-024-03130-3 38871006 PMC11485229

[2] Mukadi-Bamuleka D, Kinganda-Lusamaki E, Mulopo-Mukanya N, Amuri-Aziza A, O’Toole A, et al. First imported cases of MPXV clade Ib in Goma, Democratic Republic of the Congo: implications for global surveillance and transmission dynamics. medRxiv. 2024.09.12.24313188. http://dx.doi.org/10.1101/2024.09.12.24313188

[3] Breman JG Kalisa-Ruti Steniowski MV Zanotto E Gromyko AI Arita I . Human monkeypox, 1970-79. Bull World Health Organ. 1980;58(2):165-82. 6249508 PMC2395797

[4] Foster SO Brink EW Hutchins DL Pifer JM Lourie B Moser CR Human monkeypox. Bull World Health Organ. 1972;46(5):569-76. 4340216 PMC2480784

[5] Ladnyj ID Ziegler P Kima E . A human infection caused by monkeypox virus in Basankusu Territory, Democratic Republic of the Congo. Bull World Health Organ. 1972;46(5):593-7. 4340218 PMC2480792

[6] Van Dijck C Hoff NA Mbala-Kingebeni P Low N Cevik M Rimoin AW Emergence of mpox in the post-smallpox era-a narrative review on mpox epidemiology. Clin Microbiol Infect. 2023;29(12):1487-92. 10.1016/j.cmi.2023.08.008 37574113

[7] Okwor T Mbala PK Evans DH Kindrachuk J . A contemporary review of clade-specific virological differences in monkeypox viruses. Clin Microbiol Infect. 2023;29(12):1502-7. 10.1016/j.cmi.2023.07.011 37507009

[8] Ulaeto D Agafonov A Burchfield J Carter L Happi C Jakob R New nomenclature for mpox (monkeypox) and monkeypox virus clades. Lancet Infect Dis. 2023;23(3):273-5. 10.1016/S1473-3099(23)00055-5 36758567 PMC9901940

[9] Thornhill JP Barkati S Walmsley S Rockstroh J Antinori A Harrison LB Monkeypox virus infection in humans across 16 countries - April-June 2022. N Engl J Med. 2022;387(8):679-91. 10.1056/NEJMoa2207323 35866746

[10] World Health Organization (WHO). 2022-24 Mpox (monkeypox) outbreak: global trends. Geneva: WHO; 2024. Available from: https://worldhealthorg.shinyapps.io/mpx_global

[11] Tarín-Vicente EJ Alemany A Agud-Dios M Ubals M Suñer C Antón A Clinical presentation and virological assessment of confirmed human monkeypox virus cases in Spain: a prospective observational cohort study. Lancet. 2022;400(10353):661-9. 10.1016/S0140-6736(22)01436-2 35952705 PMC9533900

